# Blast2Fish: a reference-based annotation web tool for transcriptome analysis of non-model teleost fish

**DOI:** 10.1186/s12859-020-3507-9

**Published:** 2020-05-04

**Authors:** Chun-Hsi Tso, Jen-Leih Wu, Ming-Wei Lu

**Affiliations:** 1 Department of Aquaculture, National Taiwan Ocean University, No.2, Beining Rd., Zhongzheng Dist, Keelung City, 20224 Taiwan; 2 Institute of Cellular and Organismic Biology, Academia Sinica, Taipei, Taiwan

**Keywords:** RNA-Seq, Transcriptome, Non-model organism, Functional enrichment analysis

## Abstract

**Background:**

Transcriptome analysis by next-generation sequencing has become a popular technique in recent years. This approach is quite suitable for non-model organism study, as de novo assembly is independent of prior genomic sequences of organisms. De novo sequencing has benefited many studies on commercially important fish species. However, to understand the functions of these assembled sequences, they still need to be annotated with existing sequence databases. By combining Basic Local Alignment Search Tool (BLAST) and Gene Ontology analysis, we were able to identify homologous sequences of assembled sequences and describe their characteristics using pre-defined tags for each gene, though the above conventional annotation results obtained for non-model assembled sequences was still associated with a lack of pre-defined tags and poorly documented records in the database.

**Results:**

We introduced Blast2Fish, a novel approach for performing functional enrichment analysis on non-model teleost fish transcriptome data. The Blast2Fish pipeline was designed to be a reference-based enrichment method. Instead of annotating the BLAST single top hit by a pre-defined gene-to-tag database, we included 500 hits to search related PubMed articles and parse biological terms. These descriptive terms were then sorted and recorded as annotations for the query. The results showed that Blast2Fish was capable of providing meaningful annotations on immunology topics for non-model fish transcriptome analysis.

**Conclusion:**

Blast2Fish provides a novel approach for annotating sequences of non-model fish. The reference-based strategy allows annotation to be performed without pre-defined tags for each gene. This method strongly benefits non-model teleost fish studies for gene functional enrichment analysis.

## Background

Transcriptome analysis by next-generation sequencing (NGS), namely, RNA sequencing (RNA-Seq), has become popular in recent years. The RNA-Seq approach is a superior strategy compared to microarrays, which involve sequence-based transcriptome analysis. RNA-Seq can be carried out without a reference genomic sequence during transcriptome analysis by using de novo assembly. Take teleost fish for example, non-model fish usually lack reference genomic sequences, therefore de novo transcriptome sequencing has largely been applied in studies on commercially important fish species [1]. In our previous study, we utilized RNA-Seq to investigate endoplasmic reticulum stress in a betanodavirus-infected grouper cell line [2]. We also performed transcriptome analyses using RNA-Seq for in vivo infected samples to elaborate on the persistent infection model [3] and metamorphic development [4] of grouper. These transcriptome analyses provided valuable insight into the mechanisms governing non-model organisms.

The most popular application of RNA-Seq is transcriptome characterization and gene expression profiling in non-model organisms [5]. The major reason for this popularity is that de novo assembled sequence databases can be used as reference sequence source for further study. Therefore, NGS-based transcriptomic characterization becomes a very useful tool for non-model study. However, although sequence assembly and transcript counting may be independent of reference sequences, the assembled sequences still need to be annotated with existing sequence databases, such as GenBank, Ensembl and UniProt, for functional annotation. In the current mainstream RNA-Seq pipeline, Basic Local Alignment Search Tool (BLAST) is one of the most commonly used tools to identify homologous sequences of de novo assembled sequences [6]. Nonetheless, BLAST results for non-model assembled sequences are still associated with a lack of matched sequence records in databases. Even if a matched record exists, most functional enrichment analysis tools are not able to utilize these non-model records. For example, the important annotation tool Gene Ontology (GO) [7] has become widely adopted in the life sciences, yet most of the associations between tags and genes are based on model organisms. One common solution to this problem is to use the BLAST database of model organisms, such as mice or zebrafish, and then transfer the GO annotations from the best homolog hits. However, this process introduces a high degree of uncertainty to the results. Indeed, the evolutionary distance between the query and model sequence record will limit the reliability and sensitivity of the functional annotation process. Therefore, a superior pipeline was recently proposed that uses multiple model species in GO annotations to improve the efficiency and accuracy of annotation results [8]. Regardless, the transfer of GO labels between species eliminates the possibility of properly annotating a non-model species-specific gene. For example, there are many similarities between fish and mammals with regard to Toll-like receptor (TLR) signalling systems, but TLR13, − 19, − 20, − 21, − 22, − 23, and − 26 were later found to be members of a fish-specific TLR family [9]. Such species-specific genes would be difficult to annotate properly with a pre-defined database based on model organism studies. Moreover, non-fish gene records may bias the results of teleost studies. Therefore, using a teleost-specific annotation source should benefit annotation in this circumstance.

In this study, we developed a new approach, referred to as Blast2Fish, to establish a sensitive and efficient pipeline to perform functional enrichment analysis on non-model teleost fish. This approach does not use a pre-defined gene characterization database, such as GO. Blast2Fish instead collects published references that are linked to highly matched sequence records from BLAST results. For each PubMed article, the National Library of Medicine (NLM) assigns several terms called Medical Subject Headings (MeSH) to describe the article. We extracted MeSH from those references as annotations. Blast2Fish sorts all MeSH terms by frequency of occurrence for each transcript. The results showed that Blast2Fish provides useful terms, many of which were consistent with our previous GO-based study. The database restricted to the taxonomic node of teleost fish also largely reduced the query time against the nr (non-redundant) database and provided more species-related annotations for teleost study. Here, we employ the RNA-Seq dataset for Malabar grouper to demonstrate that a reference-based strategy is a capable approach for functional enrichment in teleost fish studies.

## Implantation

### Workflow

**Fig. 1:**
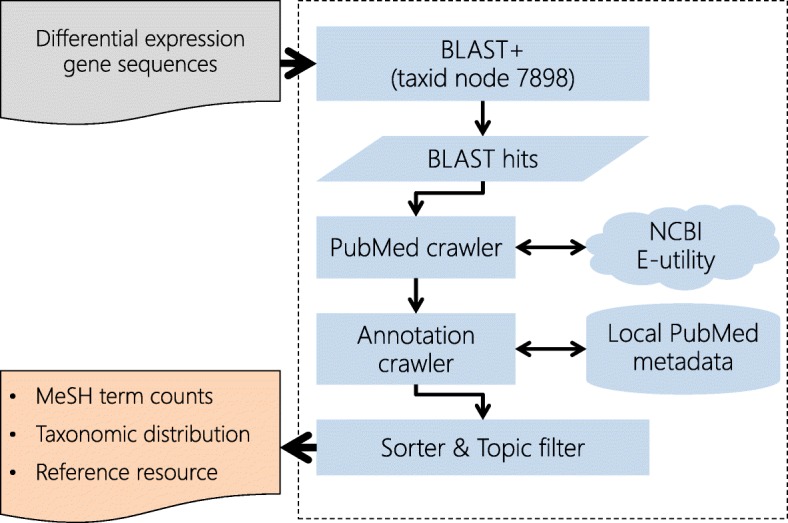
Workflow outline of Blast2Fish. This illustration briefs the workflow of Blast2Fish. The dotted line frame means the Blast2Fish system. The pipeline starts from the gene sequences file. Blast2Fish outputs MeSH term, taxonomic distribution and reference resource

An annotation workflow outline of Blast2Fish is illustrated in Fig. 1. This Blast2Fish pipeline was built with Python 3.6.4. The workflow starts from the FASTA sequence file uploaded by user. Once the web server receives and verifies the sequence file, Blast2Fish starts the BLAST search job and produces a hit table of protein sequences. After the BLAST job is complete, Blast2Fish searches for PubMed article IDs for these protein sequences through NCBI E-utility API [10]. Blast2Fish further uses article IDs to retrieve metadata, such as MeSH terms and journal names, from our local PubMed database. Finally, Blast2Fish returns sorted annotation results on web interface.

### Homologous gene searching

All query sequences in FASTA format were input to blastx from BLAST+ v2.8.1 developed by the National Center for Biotechnology Information (NCBI). The task mode was set to blastx-fast, and the expect value (E-value) cut-off was set to 1e-6. To reduce the computation resources and aim annotation in fish studies, the BLAST database used was a modified version of the nr database, namely, bonyfish_7898. The bonyfish_7898 database was built by using blastdbcmd and makeblastdb to extract bonyfish sequences from the nr database (database date: May 17, 2019). The bony fish sequence ID list was produced by TaxonKit [11]. A total of 39,930 taxonomy IDs (taxid) under node 7898 were included. The taxid node 7898 is for Actinopterygii, or ray-finned fishes in the GenBank common name. This taxonomy node covers most bony fishes [12]. To process the large dataset more efficiently, many researchers reduce the BLAST output by setting the parameter max_target_seqs to a lower number [13]. According to the manual, the max_target_seqs, maximum target sequences option controls the number of aligned sequences to keep. However, with this approach, BLAST may generate incomplete results, leading to confusion [14]. In our approach, Blast2Fish sets max_target_seqs to 500 to mitigate the issue. On the other hand, Blast2Fish relies heavily on the metadata of sequence records. Sometimes, the best BLAST hit is not well documented or lacks for reference. In this circumstance, setting a higher value of max_target_seqs helps the annotation process to retrieve more PubMed articles for a single query. To test the effect of BLAST depth, the BLAST parameter max_target_seqs was set from 1 to 500 for comparison purposes.

### References crawler and annotation record building

After BLAST finished searching, the results were outputted in a tabular format file. Blast2Fish parsed the output file to extract the query label, protein accession ID and identity. The extracted protein accession ID were used to fetch the PubMed ID (PMID) of articles by Entrez links (ELink) through the NCBI E-utility API. The maximum protein accession IDs used in the annotation were the top 500 BLAST hits for each query. With more BLAST hits, it’s expected that the identity goes lower. Especially when running non-model data, the hits are mostly across species. The interspecific gene records still provide valuable information for annotation. However, hits with identity lower than 40% were considered too low and discarded in the process. The collected PMID from the E-utility API was then used to search the corresponding metadata of articles including journal name, publication year, MeSH major terms (the terms were marked as major topics) and MeSH minor terms (the rest of terms other than major terms) in our local database. The local PubMed database was built from PubMed 2019 baseline [15] (PMID 1–29,715,642, total 29,137,780 articles). All types of MeSH terms found in the related articles were cached during the search. After all the articles were parsed, correlation scores were calculated to represent the correlation between MeSH term and query. Because popular gene produces more MeSH terms with more published articles. To normalize this effect, the term count was divided by the article counts for each query. The normalized MeSH term count was defined as correlation score. It should be noted that correlation scores are not for statistic test. All collected MeSH are linked to query by reference. The scores are used to estimate the importance between terms. Finally, Blast2Fish sorted all the MeSH terms by the sum of scores and produced top lists to show the most related terms. The lists were later visualized on the web interface. In addition, immune system is one of the most popular topics in teleost fish transcriptome study. To focus on immune system response, an additional copy of the data was generated to sort the terms under MeSH category number A15 (Hemic and Immune Systems) and G12 (Immune System Phenomena) only.

### Real RNA-Seq data annotation demonstration

In this study, we used the RNA-Seq data from our previously published study on grouper [3] for demonstration. The data used included de novo assembled transcript sequences and a significant differential expression gene list. All transcript sequences considered to be significantly differentially expressed genes were extracted to a file in FASTA format. This file was then uploaded to Blast2Fish through the web browser for a trial run to demonstrate the annotation pipeline.

### Web interface implementation

Blast2Fish is freely accessible through the web interface [8] using common web browsers. The webserver was built on Flask framework 1.0.2 with Python 3.6.4. When a user uploads a sequence file in FASTA format, annotations are run by the batch queue in the server. The user is redirected to a page containing a brief information table. The “Job ID” is automatically generated based on the MD5 hash of the uploaded file. Therefore, each ID is unique for each query job and may be used to retrieve the annotation result. If duplicated files are uploaded, the user is redirected to the same page. After the query job is complete, the results page contains MeSH keyword tables and bar charts, a reference source distribution table and a highly matched taxonomic hits distribution table.

## Results and discussion

**Fig. 2:**
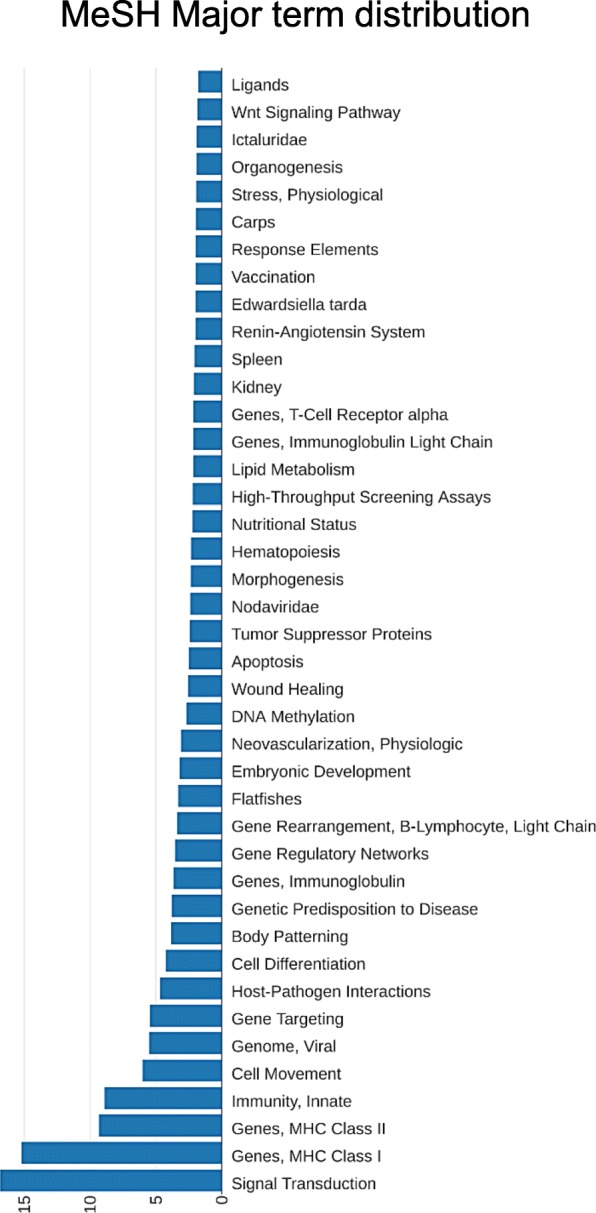
The distribution of the top 40 major MeSH of demo analysis. The visualization output of annotated major MeSH counts on the result page

We performed a demonstration using a real RNA-Seq dataset. The results are also available to users online by inputting job ID “4164553ab48d3c12d05845af87094c24” and retrieving on the home page. Table 1 shows the top 20 major MeSH terms. The first term is “Signal Transduction”, which is consistent with our GO analysis in a previous study [3]. It is already known that signal transduction plays an important role in the immune response against betanodavirus infection [16]. As shown in Fig. 2, it can be easily illustrated how signal transduction is significant in the distribution. The other terms were reasonable for describing genes in a virus-infected sample, from general terms such as “Immunity, Innate” and “Host-Pathogen Interactions” to more specific terms such as “Genes, Immunoglobulin” and “Genome, Viral”. In addition, terms such as “Cell Differentiation” and “Cell Movement” are applicable to describing T-cell activity in betanodavirus persistent infection [3]. It is worth noting that “Apoptosis” was identified in the list. This result indicated that numerous significant differentially expressed genes were considered to be apoptosis related. This finding was also consistent with our findings in a previous study [2]. Table 2 shows that the term “Signal Transduction” was again listed top 1 in the minor MeSH terms. The “Fish Diseases” also precisely matched the characterization of an infected fish sample. The “Brain” and “Nerve Tissue Proteins” are sensible for describing that our RNA sample was from the grouper brain.

**Table 1:** Top 20 MeSH major terms of the demonstration analysis

MeSH terms	Score	Annotated queries
Signal Transduction	16.80	127
Genes, MHC Class I	15.19	56
Genes, MHC Class II	9.31	54
Immunity, Innate	8.91	75
Cell Movement	6.01	38
Genome, Viral	5.50	6
Gene Targeting	5.44	10
Host-Pathogen Interactions	4.68	20
Cell Differentiation	4.26	34
Body Patterning	3.84	35
Genetic Predisposition to Disease	3.80	26
Genes, Immunoglobulin	3.68	31
Gene Regulatory Networks	3.55	24
Gene Rearrangement, B-Lymphocyte, Light Chain	3.38	17
Flatfishes	3.31	38
Embryonic Development	3.18	34
Neovascularization, Physiologic	3.10	22
DNA Methylation	2.69	17
Wound Healing	2.56	15
Apoptosis	2.51	23

**Table 2:** Top 10 MeSH minor terms of the demonstration analysis

MeSH terms	Score	Annotated queries
Signal Transduction	76.74	385
Fish Diseases	72.10	309
Brain	37.89	241
Liver	37.82	241
Transcription Factors	34.39	216
Cell Differentiation	34.35	204
Nerve Tissue Proteins	29.67	162
*Oncorhynchus mykiss*	27.38	183
Cell Movement	26.97	183
Carrier Proteins	25.72	123

To further focus the annotation on the immune system, the user can switch to the immune system-specific results page by pressing the tab button. Table 3 provides the top 10 immune system-specific MeSH major terms, such as “Major Histocompatibility Complex”, “Genes, MHC Class I” and “Genes, MHC Class II”, which are well known to play crucial roles in pathogenic disease resistance [17, 18] in grouper. These results suggest that the significantly differentially expressed genes in this RNA sample are highly related to terms such as signal transduction, apoptosis, fish disease, and antigen presentation. These terms well describe the status of betanodavirus-infected fish, even in more detail than the GO-based annotation in our previous study [3].

**Table 3:** Top 10 immune system-specific major MeSH terms of demo analysis

MeSH terms	Score	Annotated queries
Genes, MHC Class I	15.19	56
Genes, MHC Class II	9.31	54
Immunity, Innate	8.91	75
Genes, Immunoglobulin	3.68	31
Gene Rearrangement, B-Lymphocyte, Light Chain	3.38	17
Spleen	2.08	28
Immune Evasion	1.50	2
Adaptive Immunity	1.40	15
Disease Resistance	1.00	1
Major Histocompatibility Complex	0.84	15

**Fig. 3:**
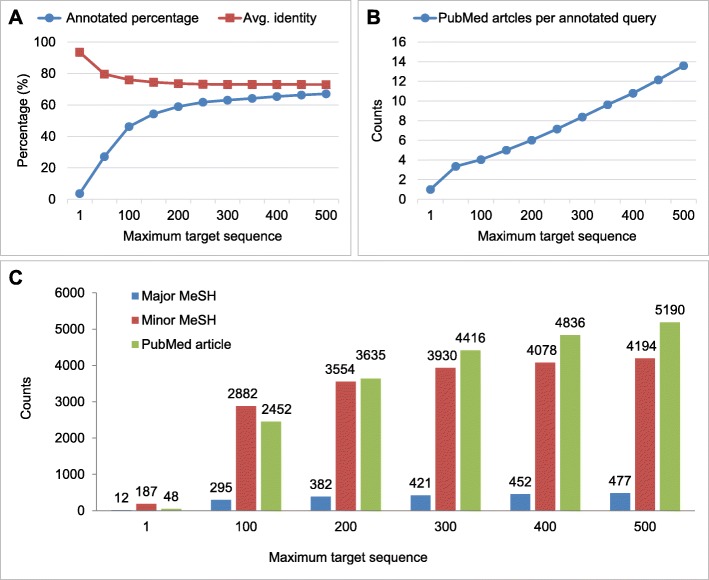
The effect of increasing parameter max_target_seqs in the annotation. **a** With increasing parameter max_target_seqs, the percentage of annotated hits rose and the average identity of BLAST hits showed a decline. **b** The counts of PubMed articles per annotated query increased by maximum target sequence setting. **c** Using more hits resulted in higher MeSH terms and PubMed articles than with the conventional method, which only uses a single best BLAST hit per query

In addition to the annotation pipeline demonstration, we investigated the effects of different BLAST depths on the nr database. As depicted in Fig. 3a, the percentage of annotated hits rose with increasing parameter max_target_seqs in the BLAST execution, and the increase slowed after the parameter exceeded 100 sequences. However, the percentage continued to grow to 67% at 500 sequences. Such an increase in annotation coverage by increasing max_target_seqs can be problematic. In Fig. 3a, the average identity of BLAST hits was 93.5% when using 1 max_target_seqs in BLAST, exhibiting a steady decline to 72.9% when max_target_seqs was set at 500 sequences. An overly low identity of hits would compromise the credibility of BLAST results. This result suggests that a rising max_target_seqs setting to obtain higher annotation coverage should be restricted to an appropriate range. In Fig. 3b, the increase of PubMed article counts per annotated query slowed between 50 to 100 max_target_seqs. And the counts steadily increased to 13.5 articles at 500 sequences. Compare with Fig. 3a, it indicated that most of the articles were contributed from already annotated queries after 200 sequences. We observed similar results in Fig. 3c. Compare with the single sequence setting, the 100 sequences setting brought great MeSH counts. In contrast, the increase of the MeSH count from 200 to 500 sequences was much smaller. These results suggested the efficiency of annotating new queries could be reduced with a high max_target_seqs setting. The top 20 major MeSH term of maximum target sequence setting 1–500 were listed in Additional file 1. Our results suggested max_target_seqs 200 to 500 seems a reasonable range of BLAST depth setting for Blast2Fish annotation.

In implementing Blast2Fish, we reconstructed the nr BLAST database to bony fish-specific databases. This downsizing had obvious benefits for the annotation pipeline. One benefit is excluding any non-fish hits to make annotation results more related to bony fish than other orthologous species. Another practical reason is that the downsized database functions more efficiently. In our demonstration, the BLAST runtime was reduced by approximately 260 times compared with the original nr database. The requirements of system memory and disk space were also considerably reduced (Table 4). Although Blast2Fish only currently provides the filter of immunology topics, it is possible to add more filters in the future. It should be noted that Blast2Fish mainly uses MeSH terms of articles and that there are some limits and biases in the MeSH indexing system. Directly utilizing the content of articles would be a better way to extract appropriate terms to describe genes. Such approach requires a natural language processing model to facilitate the recognition of proper biological terms from article content, and developing this model is an objective of our future work.

**Table 4:** The requirements of the system for performing Blast2Fish annotation

	nr DB	bonyfish_7898 DB
BLAST runtime (minutes)	50,267	**193**
BLAST memory required (GB)	57	**2.6**
Database size (GB)	199	**7.1**

## Conclusions

RNA-Seq-based transcriptome analysis is becoming a popular approach to conduct gene expression profiling on non-model organisms. However, the lack of pre-defined tags for genes in the functional enrichment process limits the reliability and sensitivity of annotation results. Blast2Fish involves a reference-based approach, which parses biological terms from published articles. To extract as many terms as possible, Blast2Fish retrieves articles from 500 BLAST hits for each query. In this study, we developed Blast2Fish and performed a demonstration using a betanodavirus-infected grouper fish brain sample. The results showed that Blast2Fish with the reference-based strategy is capable of providing meaningful annotations.

### Availability and requirements

Project name: Blast2Fish.

Project home page: http://blast2fish.ntou.edu.tw

Operating system(s): Platform independent.

Programming language: Python, JavaScript.

Other requirements: None.

License: FreeBSD.

Any restrictions to use by non-academics: Please contact authors for commercial use.

## Supplementary information

**Additional file 1.** The top 20 major MeSH term of different maximum target sequence settings. The top 20 major MeSH term of maximum target sequence setting 1–500 were listed in the file.

## Data Availability

The datasets generated and/or analysed during the current study are available on the Blast2Fish website. The job ID is “4164553ab48d3c12d05845af87094c24” and the direct link is http://blast2fish.ntou.edu.tw/job/?job_id=4164553ab48d3c12d05845af87094c24.

## Abbreviations

GO: Gene Ontology
BLAST: Basic local alignment search tool
RNA-Seq: RNA sequencing
MeSH: Medical subject headings
NGS: Next-generation sequencing
PMID: PubMed ID
ELink: Entrez links
taxid: Taxonomy ID
TLR: Toll-like receptor
